# Excitatory and Inhibitory Signaling in the Nucleus Accumbens Encode Different Aspects of a Pavlovian Cue in Sign Tracking and Goal Tracking Rats

**DOI:** 10.1523/ENEURO.0196-23.2023

**Published:** 2023-09-06

**Authors:** Kyle Duffer, Zachary S. Gillis, Sara E. Morrison

**Affiliations:** Department of Neuroscience, University of Pittsburgh, Pittsburgh, PA 15260

**Keywords:** devaluation, extinction, nucleus accumbens, Pavlovian, reward, sign tracking

## Abstract

When a Pavlovian cue is presented separately from its associated reward, some animals will acquire a sign tracking (ST) response – approach and/or interaction with the cue – while others will acquire a goal tracking response – approach to the site of reward. We have previously shown that cue-evoked excitations in the nucleus accumbens (NAc) encode the vigor of both behaviors; in contrast, reward-related responses diverge over the course of training, possibly reflecting neurochemical differences between sign tracker and goal tracker individuals. However, a substantial subset of neurons in the NAc exhibit inhibitory, rather than excitatory, cue-evoked responses, and the evolution of their signaling during Pavlovian conditioning remains unknown. Using single-neuron recordings in behaving rats, we show that NAc neurons with cue-evoked inhibitions have distinct coding properties from neurons with cue-evoked excitations. Cue-evoked inhibitions become more numerous over the course of training and, like excitations, may encode the vigor of sign tracking and goal tracking behavior. However, the responses of cue-inhibited neurons do not evolve differently between sign tracker and goal tracker individuals. Moreover, cue-evoked inhibitions, unlike excitations, are insensitive to extinction of the cue-reward relationship. Finally, we show that cue-evoked excitations are greatly diminished by reward devaluation, while inhibitory cue responses are virtually unaffected. Overall, these findings converge with existing evidence that cue-excited neurons in NAc, but not cue-inhibited neurons, are profoundly sensitive to the same behavior variations that are often associated with changes in dopamine release.

## Significance Statement

Many neurons in the nucleus accumbens (NAc) are excited by environmental cues that predict reward, while others are inhibited by such cues. Cue-excited neurons closely track animals’ behavioral responses, and even encode a specific form of learning used by sign trackers (ST) – individuals who tend to approach cues – who are prone to impulsivity and addiction-related behavior. Here, we show that cue-excited and cue-inhibited neurons seem to be involved in learning about cues, but only cue-excited neurons flexibly change their responses when cues no longer signal reward (extinction) or when the reward is no longer desired. These findings imply that different aspects of cue-reward learning are supported by different populations of NAc neurons defined by specific activity patterns.

## Introduction

Cues that are repeatedly associated with reward can acquire incentive salience: the ability to elicit motivated approach and interaction (Berridge, 2004). Notably, people and nonhuman animals vary widely in the degree to which such cues influence their behavior. In the behavior known as sign tracking (ST), animals approach and interact with a reward-associated cue (e.g., an extended lever) even when it is not located at the site of reward (Hearst and Jenkins, 1974); in contrast, other animals display the behavior known as goal tracking (GT), in which they approach the location of reward delivery rather than the cue (Boakes, 1977). A predisposition toward ST has been linked with various forms of impulsivity, risk-taking, and addiction-related behaviors, including drug-seeking and relapse (Tomie et al., 2008; Saunders and Robinson, 2013).

A considerable body of literature suggests that ST and GT involve different profiles of neurophysiological and neurochemical activity. In particular, sign tracking, but not goal tracking, is dependent on dopamine in the nucleus accumbens (NAc; Saunders and Robinson, 2012), and the profile of NAc dopamine release in sign tracker, but not goal tracker, individuals appears to reflect a reward prediction error signal (Flagel et al., 2011) thought to be used for model-free learning (Clark et al., 2012; Huys et al., 2014). We have previously shown that neural signaling in the NAc also reflects this pattern: reward-evoked excitatory responses show a gradual decrease during learning in sign tracker but not goal tracker subjects (Gillis and Morrison, 2019). On the other hand, cue-evoked excitatory responses encode the vigor (probability, rapidity, and intensity) of both sign-tracking and goal-tracking behavior. This is consistent with earlier findings regarding the role of NAc signaling in cue-directed behavior in both Pavlovian and instrumental contexts (Day et al., 2006; McGinty et al., 2013; Morrison and Nicola, 2014; Morrison et al., 2017).

There is substantial evidence that NAc dopamine is necessary for invigorating approach to reward-related targets (Nicola et al., 2005; Chow et al., 2016), and that it does so by enhancing excitatory responses to cues that are paired with reward (du Hoffmann and Nicola, 2014). On the other hand, inhibitory responses in the NAc, which are exhibited by 20–30% of neurons (Morrison et al., 2017), are far less sensitive to dopaminergic manipulation: blockade of either D1 or D2 dopamine receptors in the NAc strongly attenuates cue-evoked excitations but not inhibitions (du Hoffmann and Nicola, 2014). Because sign tracker and goal tracker individuals are thought to have different patterns of dopamine release (Flagel et al., 2011), this led us to ask whether we would see different patterns of activity in cue-excited neurons (over 50% of the population; Morrison et al., 2017) versus cue-inhibited neurons in animals performing ST or GT behavior. We also wondered whether behavioral manipulations that are known to affect NAc dopamine release, such as extinction (Sunsay and Rebec, 2014) and reward devaluation (Aitken et al., 2016; Papageorgiou et al., 2016; Keiflin et al., 2019), would have a divergent impact on the two neuronal populations.

In order to address these questions, we combined an existing data set (Gillis and Morrison, 2019) with additional single-unit recordings in the NAc during the acquisition, maintenance, and extinction of sign tracking and goal tracking behavior. This allowed us to amass a large enough population of cue-inhibited neurons for analysis. Based on previous findings during instrumental tasks (Morrison et al., 2017), we hypothesized that cue-inhibited NAc neurons, like cue-excited neurons, would encode the vigor of sign tracking and goal tracking behavior; but that the reward responses of cue-inhibited neurons, unlike cue-excited neurons, would show little change over the course of learning in sign tracker individuals. Similarly, we hypothesized that manipulations such as cue extinction and/or reward devaluation would selectively impact cue-evoked excitatory responses, but not inhibitory responses.

## Materials and Methods

All animal procedures were performed in accordance with the University of Pittsburgh animal care committee’s regulations.

### Subjects

Subjects were 11 male Long–Evans rats obtained from Charles River Laboratory. Rats weighed 275–325 g on arrival and were pair-housed until surgery (see below). Rats were maintained on a 12/12 h reverse light/dark cycle with lights on at 7 P.M.; all experiments were performed during the dark period. Rats were allowed to acclimate to the housing colony for at least 5 d and were then gently handled over at least two sessions before surgery. Food was provided *ad libitum* until 2 d before the start of behavioral training, at which time subjects were mildly food-restricted (15 g/d). Water was provided *ad libitum* throughout. Rats were weighed regularly and provided with extra food if necessary to ensure they maintained 90% of prerestriction body weight.

### Electrode arrays and surgery

Electrode arrays were constructed in house and consisted of eight Teflon-insulated tungsten microwires (A-M Systems) arranged in either a circular pattern (1 mm in diameter) or loose bundle. Electrodes were hand cut to achieve an impedance of 90–110 KΩ. We implanted arrays bilaterally in a fixed position targeted at the NAc core (AP: +1.4; ML: ± 1.5; DV: −7.0–7.2 from dura). Rats were anesthetized using isoflurane (4% for induction, 1–2% for maintenance) and treated with ketoprofen (5 mg/kg) during surgery, then provided with either ketoprofen (5 mg/kg, s.c.) or Tylenol in their drinking water for 3 d following surgery. Animals were allowed to recover for at least 7 d before the commencement of behavioral training.

### Histology

Electrode sites were labeled by passing direct current through each electrode (75 μA for 10 s) while rats were deeply anesthetized with chloral hydrate or pentobarbital. Animals were then transcardially perfused with 0.9% saline followed by 10% buffered formalin. Brains were postfixed in formalin, then transferred to 30% sucrose for at least 3 d before being sectioned at 60 μm on a cryostat. Slices were stained with cresyl violet and electrode placements were confirmed using light microscopy.

### Apparatus and behavior

All training and recording took place in the same standard operant chambers (Coulbourn Instruments), which were equipped with a house light, a speaker, and a pellet dispenser mounted above a food magazine recessed into the side wall. An infrared photodetector inside the magazine detected entries and exits. Either one or two retractable levers were present next to the food magazine (side counterbalanced between subjects), and a white cue light was mounted over each lever that was present. When two levers were present, only one was used for a particular subject. For some sessions, three nosepoke operanda were present on the opposite side of the chamber from the food magazine; these were inactive. The task was controlled by Coulbourn software (GraphicState 3.0 or 4.0).

Rats were trained on a Pavlovian conditioned approach (PCA) task as reported previously (Gillis and Morrison, 2019). Briefly, rats were initially trained to retrieve sucrose pellets (BioServ, 45 mg) from the food magazine over two daily sessions. Each session consisted of 50 pellets delivered on a variable time schedule averaging 60 s. During the second magazine training session, rats were habituated to the recording apparatus. Subsequently, rats were trained for 7 daily sessions on the PCA task, which consisted of 25 trials separated by an intertrial interval randomly selected from a truncated exponential distribution averaging 60 s. On each trial, the cue, consisting of an extended lever accompanied by a flashing cue light (5 Hz), was presented for 8 s. The cue was accompanied by an auditory signal (1 s, 500-Hz intermittent tone). After 8 s, the lever retracted, the cue light was extinguished, and a sucrose pellet was delivered to the food magazine. The rat was not required to perform any action for the reward to be delivered.

Neuronal recording took place on all 7 d of acquisition of the PCA task. After the completion of training, a subset of rats underwent recording during one to two extinction sessions. (When >1–2 d had elapsed following the last training day, rats were given 1 d of retraining before extinction.) Extinction sessions were identical to the PCA task except that no rewards were delivered. Analyses shown are for the first extinction session only.

Another subset of rats underwent a reward devaluation procedure before the extinction session. After the completion of training, these rats were given alternating days of sucrose pellets delivered in the same operant box used for recording, followed by an injection of lithium chloride (LiCl; 0.6 m, 5 ml/kg, i.p.), or exposure to the recording context only (no sucrose pellets) followed by an injection of sterile saline (5 ml/kg). Sucrose pellets were delivered on a variable time schedule averaging 30 s; rats were initially given 40 pellets, then decreasing to 30, 20, or 10 in subsequent sessions based on the amount they actually consumed. Devaluation sessions continued until rats did not eat any pellets offered (generally four to five exposures).

### Electrophysiology

Neural data were recorded using Plexon hardware and software. Rats were connected to a lightweight 16-channel headstage and either a motorized or nonmotorized commutator; both allowed freedom of movement throughout the operant box. Voltages were bandpass filtered between 220 Hz and 6 kHz, amplified 500×, and digitized at 40 kHz. Spikes were stored in 1.4 ms segments and hand-sorted using principal component analysis and visual inspection of waveform features (Offline Sorter, Plexon). Units were analyzed only if they were >75 μV, had a signal-to-noise ratio of >2:1, and had <0.1% interspike intervals <2 ms. Isolation of units was verified using autocorrelograms, as well as cross-correlograms for units recorded on the same electrode.

### Analysis of behavior

We quantified the degree to which subjects engaged in sign tracking or goal tracking using a PCA index (Meyer et al., 2012). The PCA index is a composite of three ratios, the probability index, bias index, and latency index, which all vary from −1.0 to +1.0. In each case, negative numbers indicate a preference for interacting with the food magazine (goal tracking), and positive numbers indicate a preference for interacting with the lever (sign tracking). The probability index is calculated as P_lever_ – P_magazine_, where P is probability of the indicated action. The bias index is calculated as (#lever press – #magazine entry)/(#lever press + #magazine entry). The latency index is calculated as (magazine latency – lever latency)/(cue length). For trials in which a behavior is not performed, the latency for that behavior is defined as the cue length (8 s).

### Analysis of neural data

All analyses were performed using custom-written programs in MATLAB. See Table 1 for detailed statistics. Cue-excited and cue-inhibited neurons were identified as previously described (Morrison et al., 2017; Gillis and Morrison, 2019). Briefly, we defined a Poisson distribution approximating the baseline firing rate in the 1 s before cue onset. We identified cue-excited and cue-inhibited neurons by the presence of at least three consecutive 10-ms bins in which the firing rate respectively exceeded the upper or was less than the lower 99.9% confidence interval of the baseline distribution. The time window for each inhibition was determined by identifying at least three consecutive bins in which the firing rate returned to or exceeded either the baseline firing rate or the firing rate in the 1 s before the start of the inhibition, whichever was lower. If no “inhibition off” time was identified in the 500 ms following cue onset, the duration of the inhibition was defined as 500 ms.

**Table 1:** Detailed statistics for all analyses in the manuscript

Figure/ panel	Description	*N*	Test	Statistic	*p*-value
3*E*	Vigor index (ROC analysis comparing top and bottom 50% of latency to first action)	79 cue-excited cells	Wilcoxon signed-rank test	Z = 1.25	*p* = 0.21
3*F*	Vigor index	47 cue-inhibited cells	Wilcoxon signed-rank test	Z = −1.34	*p* = 0.18
4*A*	Average cue-related activity (500 ms) on first/last day of training	45 cue-excited cells (first session), 79 cells (last session)	Wilcoxon rank-sum test	Z = 0.35	*p* = 0.72
4*B*	Average cue-related activity (custom time windows) on first/last day of training	14 cue-inhibited cells (first session), 47 cells (last session)	Wilcoxon rank-sum test	Z = −0.08	*p* = 0.94
4*C*	Proportion of excited/inhibited neurons in first/last session	92 cells (first session), 169 cells (last session)	χ^2^ test	χ^2^(2,261) = 6.32	*p* = 0.04
4*D*	Proportion of excited/inhibited neurons in first session for ST vs GT individuals	32 cells (ST), 60 cells (GT)	χ^2^ test	χ^2^(2,92) = 16.76	*p* < 0.001
4*D*	Proportion of excited/inhibited neurons in last session for ST vs GT individuals	51 cells (ST), 118 cells (GT)	χ^2^ test	χ^2^(2,169) = 3.87	*p* = 0.14
5*B*	Average reward-related activity in ST vs GT individuals (final day of training)	29 cue-excited cells from ST, 50 from GT	Wilcoxon rank-sum test	Z = −2.86	*p* = 0.004
5*D*	Average reward-related activity in ST vs GT individuals (final day of training)	12 cue-inhibited cells from ST, 35 from GT	Wilcoxon rank-sum test	Z = 0.99	*p* = 0.32
6*A*	Cue-related activity by trial number (example cue-excited cell)	25 trials	One-way ANOVA	*F*_(1)_ = 32.34	*p* < 0.001
6*B*	Cue-related activity by trial number (example cue-inhibited cell)	25 trials	One-way ANOVA	*F*_(1)_ = 1.88	*p* = 0.18
6*E*	Main effect of trial number on cue-related activity during extinction	62 cue-excited cells	Two-way ANOVA	*F*_(1,1546)_ = 125.5	*p* < 0.001
6*F*	Main effect of trial number on cue-related activity during extinction	22 cue-inhibited cells	Two-way ANOVA	*F*_(1,546)_ = 0	*p* = 0.97
6*E*	Average cue-related activity in ST and GT individuals (third 5-trial bin during extinction)	26 cue-excited cells from ST, 36 from GT	Wilcoxon rank-sum test	Z = 1.70	*p* = 0.08
6*F*	Average cue-related activity in ST and GT individuals (last 5 trials of extinction)	14 cue-inhibited cells from ST, 8 cells from GT	Wilcoxon rank-sum test	Z = −2.83	*p* = 0.005
7*B*	Interaction between devaluation status and trial number (continuous variable)	21 cue-excited cells (devaluation), 62 cells (no devaluation)	Two-way ANOVA	*F*_(1,2072)_ = 17.2	*p* < 0.001
7*B*	Average cue-related activity following devaluation or no devaluation (first 5 trials of extinction)	21 cue-excited cells (devaluation), 62 cells (no devaluation)	Wilcoxon rank-sum test	Z = 4.39	*p* < 0.001
7*C*	Interaction between devaluation status and trial number (continuous variable)	22 cue-inhibited cells (devaluation), 23 cells (no devaluation)	Two-way ANOVA	*F*_(1,1121)_ = 0	*p* = 0.96
7*C*	Average cue-related activity following devaluation or no devaluation (first 5 trials of extinction)	22 cue-inhibited cells (devaluation), 23 cells (no devaluation)	Wilcoxon rank-sum test	Z = 0.90	*p* = 0.37

If both excitatory and inhibitory responses were identified within 500 ms after cue onset, we examined the mean Z-score in 200 and 500 ms following cue onset. If both of these were positive, the neuron was categorized as cue-excited; if both were negative, the neuron was categorized as cue-inhibited. In the rare case that one was positive and one was negative, the Z-score with larger absolute value determined how the neuron was categorized.

Peristimulus time histograms (PSTHs) for individual neurons were calculated in 10-ms bins and are shown smoothed using a five-bin moving average. Population PSTHs were calculated in 10-ms bins and normalized by Z-scoring relative to baseline (1 s before cue onset) before averaging across neurons; the average activity was then smoothed using a five-bin moving average.

Analyses of individual neurons were performed using activity from the 500 ms following cue onset for cue-excited neurons, or in variable windows customized for each cue-inhibited neuron, as previously described (Morrison et al., 2017). The start of each custom window was the “inhibition on” time determined by the algorithm described above, and the end was either the “inhibition off” time determined by the algorithm or 500 ms, whichever was smaller. Among cells recorded on the last day of training, inhibition on times ranged from 10 to 180 ms (mean = 62 ms). The earliest inhibition off time was 260 ms, although most were much greater (mean = 443 ms).

In some cases, we used ROC (receiver operating characteristic) analysis to generate an index (e.g., vigor index) comparing two distributions of firing rates. For these indexes, which are derived from the area under an ROC curve, a value of 0.5 indicates that the two distributions are indistinguishable. We calculated a *p*-value for each index by randomly re-shuffling the data 1000 times (permutation test).

Within extinction sessions, we identified neurons that did or did not extinguish their cue-evoked responses using a one-way ANOVA with trial number as a continuous variable. This was applied to firing rates in the 500 ms following cue onset (cue-excited neurons) or within custom windows (cue-inhibited neurons). Extinguishing cells were defined by *p*-values < 0.01, along with a decreasing cue response (lower firing rate for cue-excited neurons, higher firing rate for cue-inhibited neurons).

## Results

**Figure 1.**
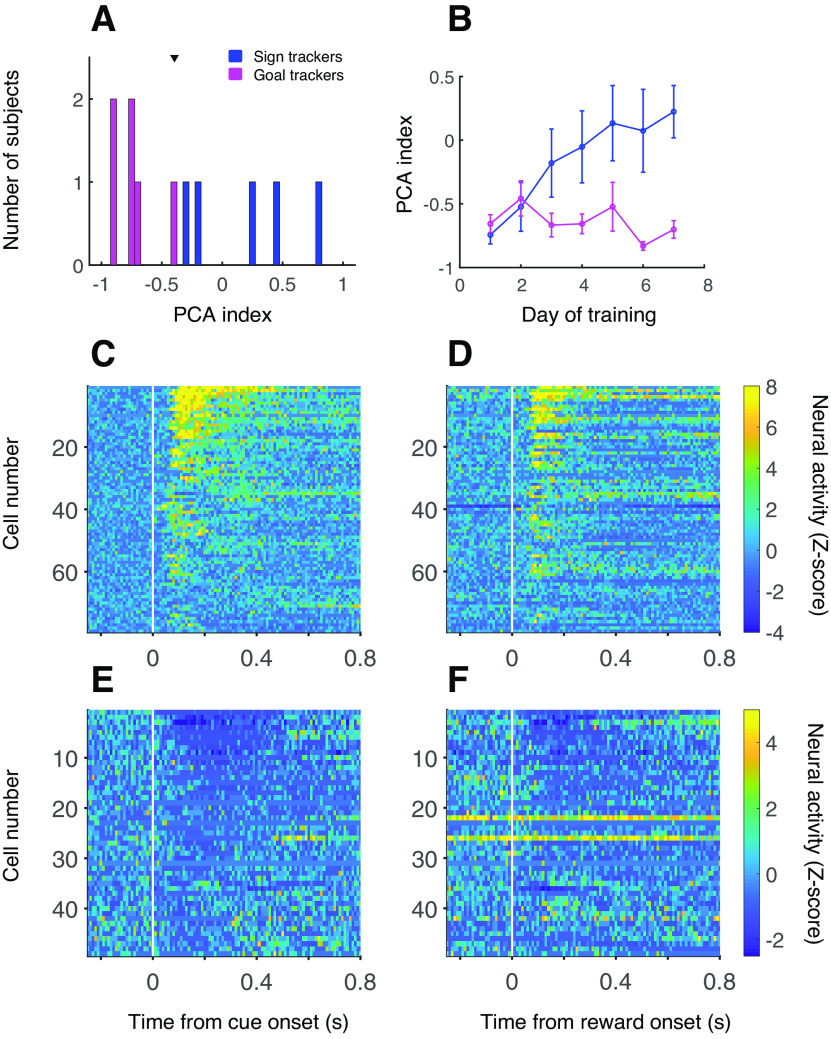
Behavior and overview of NAc neural activity. ***A***, PCA index for each subject calculated as an average over the last 3 d of training. Arrowhead, population mean. ***B***, Average PCA index over 7 d of training for sign trackers (blue) and goal trackers (magenta). Error bars, SEM. ***C–F***, Average normalized activity of neurons with an excitatory cue response (***C***, ***D***; *n* = 79) or an inhibitory cue response (***E***, ***F***; *n* = 49) around the time of cue onset (***C***, ***E***) or reward delivery (***D***, ***F***) on the last day of training. Activity is calculated in 10-ms bins with no smoothing.

We used custom-built electrode arrays to record from the nucleus accumbens (NAc) core while rats (*n* = 11) acquired a Pavlovian conditioned approach (PCA) task that typically elicits sign tracking (ST), goal tracking (GT), or a combination of these behaviors. ST behavior is represented by lever deflections, indicating interaction with the cue; GT behavior is represented by food magazine entries, indicating interaction with the site of reward. As we and others have done before (Meyer et al., 2012; Morrison et al., 2015; Gillis and Morrison, 2019), we quantified individuals’ propensity for ST versus GT behavior using a composite PCA index (see Materials and Methods). The PCA index ranges from −1.0 (only GT behavior) to +1.0 (only ST behavior). By the end of training, subjects showed a range of ST and GT behavior, as illustrated by their average PCA index over the last 3 d of training (Fig. 1*A*); however, most subjects performed some degree of GT, resulting in a PCA index distribution that was negatively skewed. Therefore, we operationally defined “sign trackers” as those subjects with a PCA index greater than the population mean (Fig. 1*A*, arrowhead), and “goal trackers” as those with a PCA index less than the mean (*n* = 5 sign trackers, *n* = 6 goal trackers). This resulted in categorizing as sign trackers only those subjects having an appreciable amount of interaction with the lever. Over 7 d of training, the PCA index of sign trackers gradually increased, while the PCA index of goal trackers stayed relatively stable (Fig. 1*B*).

### NAc excitations and inhibitions encode aspects of sign tracking and goal tracking behavior

**Figure 2.**
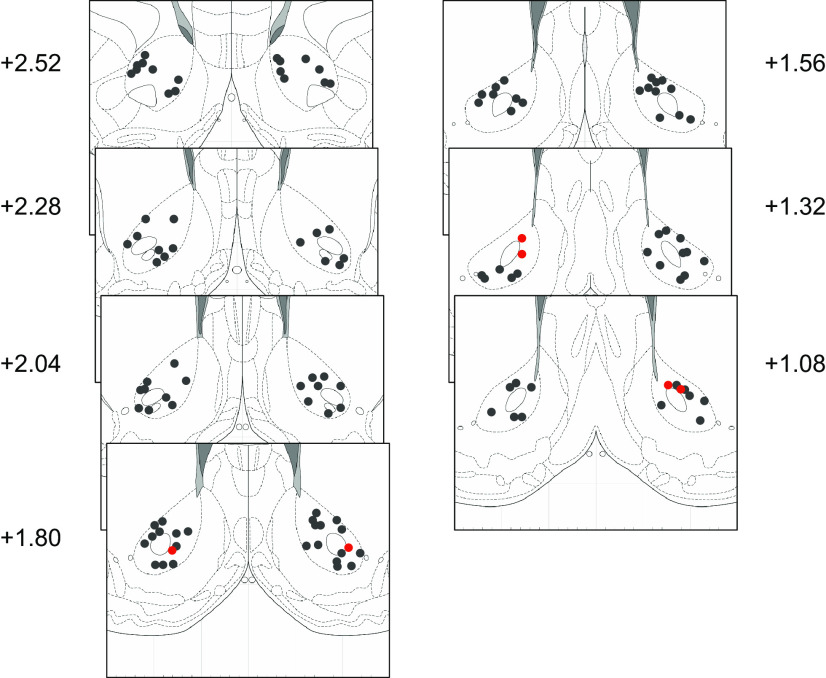
Histologic reconstruction of recording locations. Panels are coronal sections (Paxinos and Watson, 2007) showing the location of electrode tips or approximate center of electrode bundles. Locations are derived from electrolytic lesions and/or electrode tracks. Numbers are distance in mm from bregma. Red, recording locations for subjects new to the current data set. All new locations represent the center of an electrode bundle. Black, recording locations for subjects from Gillis and Morrison (2019). There are disproportionately more black dots because of the use of circular arrays rather than electrode bundles.

We recorded from 169 neurons during the final (seventh) day of training, including 122 from a data set on which we have previously reported (Gillis and Morrison, 2019). Of these neurons, 79 (47%) exhibited cue-evoked excitatory responses (58 of which were analyzed in the prior report), and 47 (28%) exhibited cue-evoked inhibitory responses, similar proportions to those found in earlier studies (Morrison et al., 2017). Of the neurons with cue-evoked excitations, 29 were recorded in sign tracker individuals and 50 in goal trackers; of the neurons with cue-evoked inhibitions, 12 were recorded in sign trackers and 35 in goal trackers. The event-related activity of cue-excited and cue-inhibited neurons is summarized in Figure 1*C–F*: both excitations and inhibitions varied in intensity but were mainly phasic and brief, although inhibitions tended to be more sustained. Notably, most neurons with cue-evoked excitations also exhibited phasic excitatory responses to reward delivery (Fig. 1*D*), while most neurons with cue-evoked inhibitions, with a couple of prominent exceptions, exhibited phasic inhibitory responses to reward delivery (Fig. 1*F*). Histologic reconstruction (Fig. 2) shows that most recorded neurons were clearly in the NAc core, although a handful may have been on the border with shell.

**Figure 3.**
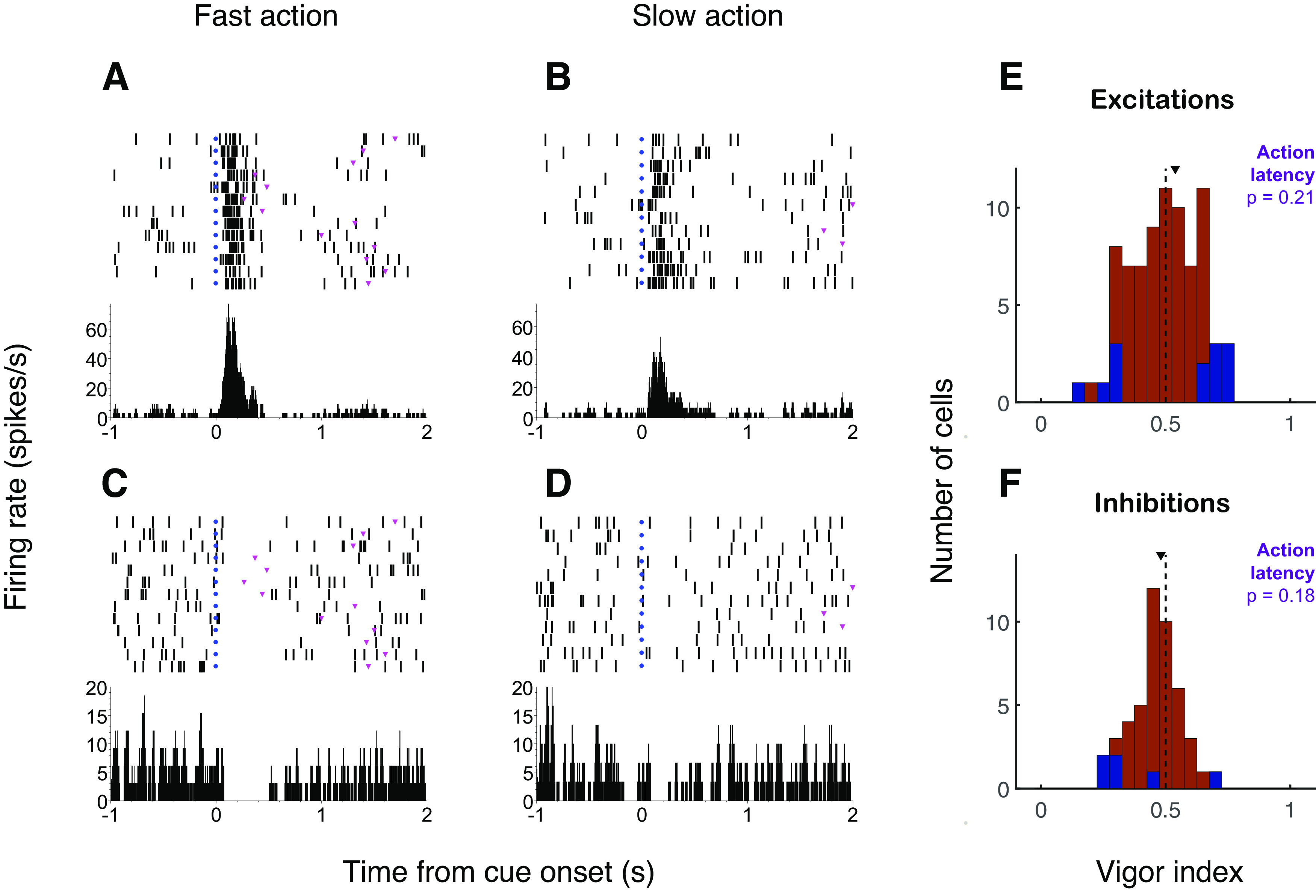
The vigor of sign tracking and goal tracking may be represented by both excitatory and inhibitory cue-evoked responses. ***A–D***, Example of a neuron that shows a stronger excitatory response (***A***, ***B***) and a neuron that shows a stronger inhibitory response (***C***, ***D***) when the cue is followed by a magazine entry with faster (***A***, ***C***) or slower (***B***, ***D***) latency. Left panels, magazine latency >50th percentile; right panels, magazine latency <50th percentile. Trials are shown chronologically with the earliest trials on top. Blue dots, cue onset. Magenta triangles, magazine entry. ***E***, ***F***, Vigor index for latency to first action (lever press or magazine entry) for all cue-excited neurons (***E***; similar to Gillis and Morrison, 2019; their Figure 3*E*) and cue-inhibited neurons (***F***). Blue represents vigor index significantly different from 0.5 (*p* < 0.05, permutation test). Arrowhead indicates population median.

We have previously shown that cue-evoked excitations encode information about the vigor – the speed and intensity – of actions directed toward a reward-associated target in both an instrumental (Morrison et al., 2017) and Pavlovian (Gillis and Morrison, 2019) context. Notably, in the case of the PCA task, cue-evoked excitations encoded the vigor of both ST and GT behavior. In an instrumental context, cue-evoked inhibitions also encoded aspects of locomotor vigor, such as motion onset latency (Morrison et al., 2017). Therefore, we first asked whether cue-evoked inhibitions encode the vigor of ST and/or GT behavior during the PCA task. In Figure 3*A–D*, two example neurons recorded from the same subject on the last day of training demonstrate that both excitatory and inhibitory responses may be related to vigor. The cue-excited neuron shown in Figure 3*A*,*B* exhibits a stronger phasic response when the subsequent actions (in this case, magazine entries) are conducted with shorter latency. Similarly, the cue-inhibited neuron shown in Figure 3*C*,*D* exhibits a deeper, more complete inhibition when the subsequent magazine entry occurs with shorter latency.

In order to quantify vigor encoding across the neuronal population, we used ROC analysis to calculate a “vigor index” comparing neural activity on trials with short latency (<50th percentile) or long latency (≥50th percentile) to first action (lever press or receptacle entry). A vigor index of 0.5 indicates no difference between neural responses in the two conditions. We previously reported that cue-excited NAc neurons encoded action latency as a population (Gillis and Morrison, 2019), indicated by a significant shift away from 0.5. In the current data set, the vigor index is no longer significantly shifted away from 0.5 (Fig. 3*E*; *p* = 0.21, Wilcoxon signed-rank test), although the shift is still significant if only GT actions are considered (*p* = 0.04; data not shown) or if a shorter time window (250 ms after cue onset) is analyzed (*p* = 0.05; data not shown). Similarly, the vigor index for cue-evoked inhibitions is not significantly shifted away from 0.5 (Fig. 3*F*; *p* = 0.18); however, a number of individual neurons have a vigor index <0.5, indicating that they are more strongly inhibited before faster actions. Overall, this is consistent with the previously observed smaller, more variable contribution of vigor to activity in cue-inhibited neurons (Morrison et al., 2017).

### Excitations and inhibitions in NAc evolve differently during acquisition of behavior

**Figure 4.**
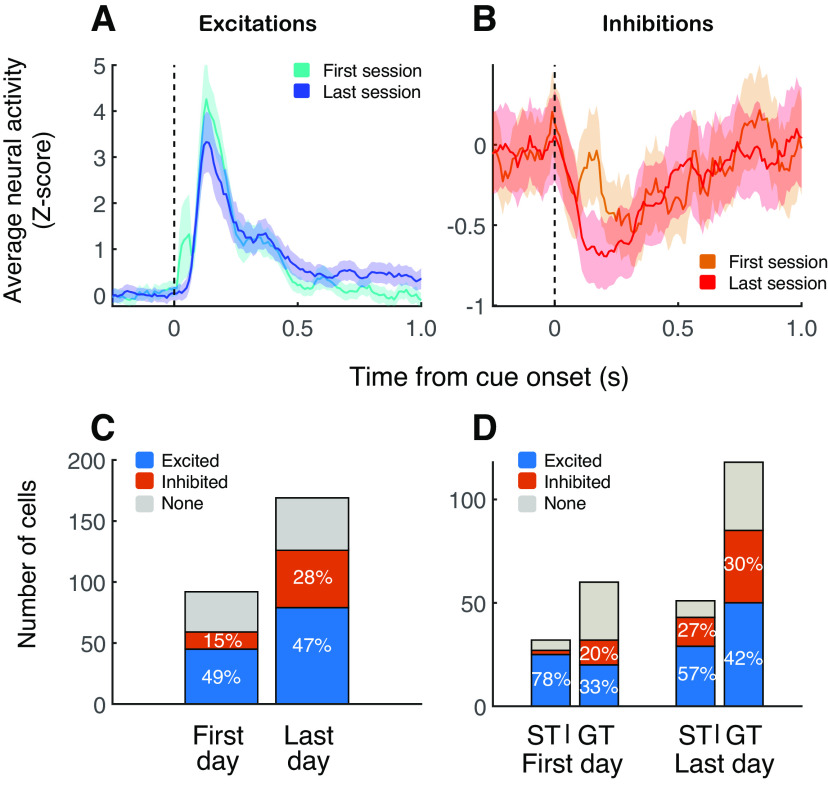
Inhibitory cue responses are strengthened over the course of training. ***A***, ***B***, Population normalized neural activity aligned on cue onset among cue-excited neurons (***A***) and cue-inhibited neurons (***B***). Cyan and orange lines, activity from first day of training; blue and red lines, activity from final (seventh) day of training. Shading indicates SEM. ***C***, Number of cue-excited (blue), cue-inhibited (orange), and non-cue responsive cells recorded on the first versus last day of training. Proportions are significantly different (χ^2^ test, *p* < 0.05). ***D***, Number of cue-excited (blue), cue-inhibited (orange), and non-cue responsive cells recorded on the first day (left) versus last day (right) of training for sign tracker and goal tracker individuals. The proportion of cue-inhibited cells for sign trackers on the first day was 6%; other values appear in figure. First day proportions are significantly different for sign trackers versus goal trackers (χ^2^ test, *p* < 0.001). Last day proportions are not significantly different (*p* = 0.16).

We next examined whether and how cue-related and reward-related excitations and inhibitions evolve over the course of training. We previously reported that cue-evoked excitations in the NAc show little change over 7 d of training in both sign trackers and goal trackers (Gillis and Morrison, 2019), consistent with the observation that cue-evoked dopamine release changes only subtly, if at all, in outbred sign tracker rats (Flagel et al., 2011). In the current data set, among cue-excited neurons, we again found little difference between population average cue-evoked activity on the first day versus the last day of training (Fig. 4*A*; *p* = 0.72, Wilcoxon rank-sum test). Among cue-inhibited neurons, cue-evoked responses strengthened over the course of training in some time windows (Fig. 4*B*), although this difference was not significant when the magnitude of inhibition was calculated in customized time windows for each neuron (*p* = 0.9). However, inhibitory responses became significantly better represented among the population, increasing from 15% to 28% of recorded neurons (Fig. 4*C*; *p* = 0.04, χ^2^ test).

The data shown in Figure 4*D* suggests some differences in the proportions of cue-excited and cue-inhibited neurons among sign tracker and goal tracker individuals. Indeed, sign trackers yielded a significantly higher proportion of cue-excited cells on the first day of training compared with goal trackers (*p* < 0.001, χ^2^ test). There was also a trend in this direction on the last day of training (*p* = 0.08, χ^2^ test for “excited” vs “nonexcited” cells), although the proportions of the three cell types were not different overall (*p* = 0.14).

**Figure 5.**
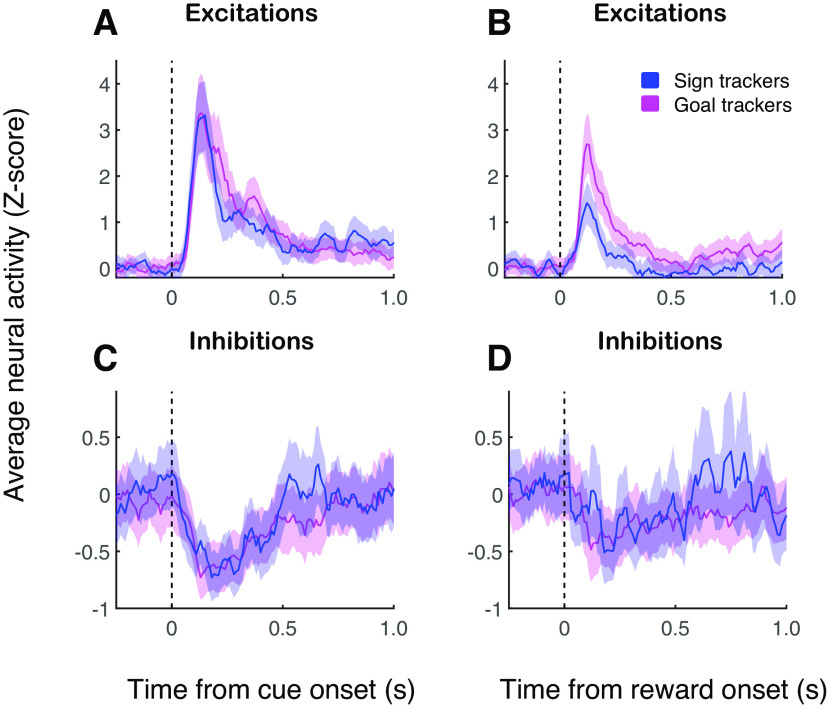
Inhibitory responses, unlike excitatory responses, do not differ between sign trackers and goal trackers after training. Population normalized neural activity aligned on cue onset (***A***, ***C***) or reward delivery (***B***, ***D***) among cue-excited cells (***A***, ***B***) and cue-inhibited cells (***C***, ***D***). Shading indicates SEM. Panels ***A***, ***B*** are similar to Gillis and Morrison (2019; their Figure 5*A,B*).

As we have demonstrated previously (Gillis and Morrison, 2019), cue-evoked excitations were similar among sign tracker and goal tracker subjects on the last day of training (Fig. 5*A*); however, excitatory responses to reward differed markedly between the two groups (Fig. 5*B*), with sign trackers showing an attenuated response compared with goal trackers (*p* = 0.004, Wilcoxon rank-sum test). This may be a downstream result of the difference in reward-evoked dopamine release between sign trackers and goal trackers (Flagel et al., 2011), which is thought to reflect differences between the groups in the use of a dopaminergic reward prediction error for model-free learning (Clark et al., 2012; Huys et al., 2014). This led us to ask whether these intergroup differences in dopamine dynamics might be reflected in cue-evoked and/or reward-evoked inhibitory responses in the NAc. On the contrary, we found little or no difference in inhibitory responses to either the cue (Fig. 5*C*) or, importantly, to reward (Fig. 5*D*), on the last day of training (reward response, *p* = 0.3). This may be related to the relative insensitivity of cue-evoked inhibitions to dopaminergic manipulations (du Hoffmann and Nicola, 2014).

### Cue-evoked excitations, but not inhibitions, are attenuated by extinction and reward devaluation

We previously reported that about half of NAc cue-evoked excitations “extinguish” in concert with behavior during an extinction session (Gillis and Morrison, 2019), although another subset retain their response to the cue even after behavior (sign tracking and/or goal tracking) is extinguished. Excitatory cue responses were slower to extinguish in sign trackers, consistent with the finding that sign tracking, compared with goal tracking, is relatively resistant to extinction (Ahrens et al., 2015). We reasoned that the attenuation of excitatory responses might be attributed, at least in part, to a reduction in NAc dopamine release in response to the extinguished cue (Sunsay and Rebec, 2014). Therefore, we wondered whether cue-evoked inhibitions, which are relatively insensitive to blockade of dopamine receptors (du Hoffmann and Nicola, 2014), might be more resistant to extinction than cue-evoked excitations.

**Figure 6.**
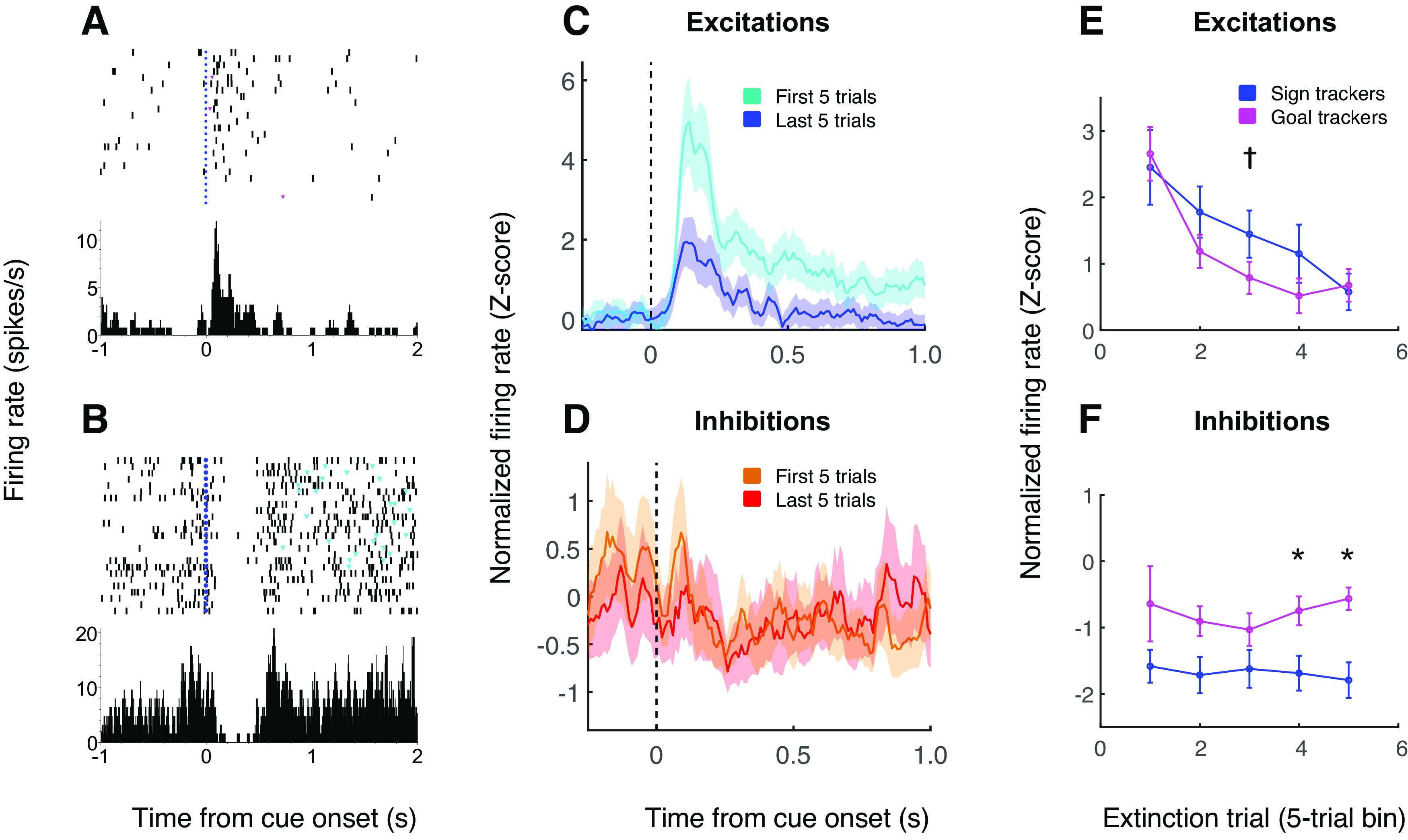
Inhibitory cue-evoked responses, unlike excitatory responses, do not extinguish in concert with behavior. ***A***, ***B***, Representative examples of a cue-excited neuron that extinguishes its response during extinction (***A***) and a cue-inhibited neuron that does not (***B***). The two neurons were recorded from the same subject during the same session. Trials are shown chronologically with the earliest trial on top. Blue dots, cue onset. Cyan triangles, lever presses. ***C***, ***D***, Population average normalized activity aligned on cue onset among cue-excited cells (***C***) and cue-inhibited cells (***D***) during the first five trials (cyan/orange) or last five trials (blue/red) of an extinction session. ***E***, ***F***, Population average normalized activity among sign trackers (blue) and goal trackers (magenta) calculated in five-trial bins over the course of extinction for cue-excited cells (***E***) and cue-inhibited cells (***F***). Panel ***E*** is similar to Gillis and Morrison (2019; their Figure 6*E*). For excitations, activity is from the first 1 s after cue onset; for inhibitions, activity is calculated in custom time windows for each cell. Error bars, SEM. Asterisk indicates activity is significantly different in sign trackers versus goal trackers (Wilcoxon rank-sum test, *p* < 0.05; dagger, *p* = 0.08).

The current data set includes 91 neurons recorded during an extinction session, including 63 that were cue-excited and 23 that were cue-inhibited. Two example cells, recorded from the same subject (a sign tracker) during the same extinction session, are shown in Figure 6*A*,*B*. The representative cue-excited cell (Fig. 6*A*) significantly decreases its firing rate over the course of 25 extinction trials (*p* < 0.001, one-way ANOVA), similar to the “extinguishing cells” that made up 46% of cue-excited neurons in a previous study (Gillis and Morrison, 2019), while the representative cue-inhibited cell (Fig. 6*B*) does not significantly decrease its response (*p* = 0.18). This pattern was evident in the population as a whole: among cue-excited cells (Fig. 6*C*), the population average response significantly decreased over the course of extinction for both sign trackers and goal trackers (two-way ANOVA, main effect of trial number, *F*_(1,1546)_ = 125.5, *p* < 0.001). As previously observed, this decrease was slightly more gradual for sign trackers than goal trackers (Fig. 6*E*). Among cue-inhibited cells (Fig. 6*D*), on the other hand, inhibitions did not decrease in magnitude, instead remaining stable over the course of extinction (two-way ANOVA, main effect of trial number, *F*_(1,546)_ = 0, *p* = 0.97). Notably, by the end of extinction, sign trackers’ inhibitory responses were stronger than those of goal trackers (Fig. 6*F*; *p* = 0.005, Wilcoxon rank-sum test).

**Figure 7.**
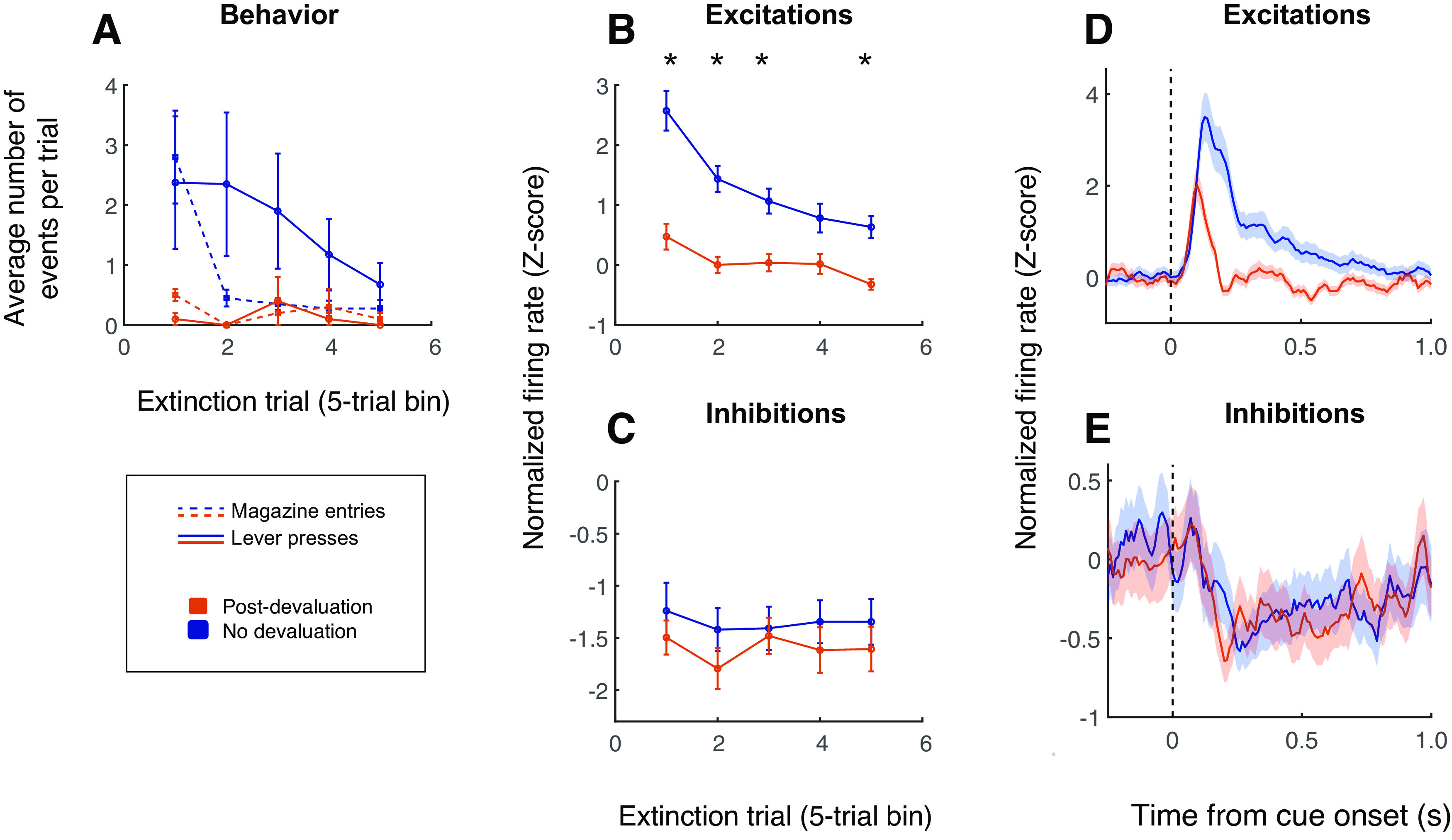
Excitatory cue responses, but not inhibitory cue responses, are sharply reduced following reward devaluation. ***A***, Behavioral responses, averaged in five-trial bins, over the course of an extinction session that is preceded by reward devaluation (orange) or is not preceded by reward devaluation (blue). Solid lines, lever presses; dashed lines, magazine entries. Error bars, SEM. ***B***, ***C***, Population average normalized activity among cue-excited cells (***B***) or cue-inhibited cells (***C***), averaged in five-trial bins, over the course of an extinction session that is preceded by reward devaluation (orange) or is not preceded by reward devaluation (blue). Error bars, SEM. Asterisks indicate activity is significantly different in devalued versus nondevalued subjects (Wilcoxon rank-sum test, *p* < 0.05). ***D***, ***E***, Population average normalized activity aligned on cue onset among cue-excited cells (***D***) or cue-inhibited cells (***E***) during an extinction session. Orange, extinction session preceded by reward devaluation; blue, extinction session not preceded by reward devaluation. Shading indicates SEM.

Finally, we examined the responses of cue-excited and cue-inhibited neurons in the NAc to cues following reward devaluation, a manipulation that has been shown to reduce dopamine release in response to cues associated with the devalued outcome (Aitken et al., 2016; Papageorgiou et al., 2016). We and others have reported that, similar to extinction, sign tracking, compared with goal tracking, is relatively insensitive to reward devaluation (Morrison et al., 2015; Keefer et al., 2020). On the other hand, some studies have shown that sign tracking can be diminished following reward devaluation under certain conditions, especially if the devaluation is conducted in the same context where the ST behavior is performed (Derman et al., 2018; Amaya et al., 2020). In the current experiment, we chose to perform a repeated “in-context” reward devaluation via taste aversion conditioning (similar to Amaya et al., 2020). Consistent with recent reports, we found that, under these conditions, both sign tracking and goal tracking responses were reduced to near zero (Fig. 7*A*).

We recorded from 46 neurons following reward devaluation; of these, 17 were algorithmically identified as cue-excited and 18 as cue-inhibited. Overall, we found that NAc excitatory responses to the cue were profoundly reduced during an extinction session preceded by reward devaluation (Fig. 7*B*) compared with an extinction session that was not (two-way ANOVA, devaluation status × trial number, *F*_(1,2072)_ = 17.2, *p* < 0.001). Cue-evoked activity was significantly impacted by devaluation within the first five trials of the session (*p* < 0.001, Wilcoxon rank-sum test). Cue-evoked inhibitions, on the other hand, were not diminished following reward devaluation (Fig. 7*C*), just as they showed little to no effect of extinction (two-way ANOVA, devaluation status × trial number, *F*_(1,1121)_ = 0, *p* = 0.96). If anything, inhibitory responses became slightly more intense; however, they were not significantly different, over the first five trials, from inhibitory responses during an extinction session that was not preceded by reward devaluation (*p* = 0.37, Wilcoxon rank-sum test). Likewise, population average activity showed that reward devaluation results in a profound decrease in cue-evoked excitation (Fig. 7*D*), but that cue-evoked inhibition remains virtually unchanged (Fig. 7*E*). Overall, NAc cue-evoked excitations closely tracked behavior as it diminished during extinction and/or reward devaluation, but cue-evoked inhibitions were uncoupled from behavior.

## Discussion

The nucleus accumbens has often been thought of as a hub for integrating motivational information about a cue or context with cognitive information, such as goal selection, and promoting the appropriate motor response (Mogenson et al., 1980; Ikemoto and Panksepp, 1999; Nicola, 2010; Morrison et al., 2017). The activity of individual neurons in the NAc has been shown to encode information about the proximity and reward value of a cue that elicits approach, along with locomotor factors such as motion onset latency and movement speed (McGinty et al., 2013; Morrison and Nicola, 2014; Morrison et al., 2017). This is true in Pavlovian as well as instrumental tasks: in the context of the Pavlovian conditioned approach task used here, cue-evoked excitatory responses encode the vigor of both sign tracking and goal tracking (Gillis and Morrison, 2019), although the two behaviors are thought to result from different learning processes (Clark et al., 2012; Huys et al., 2014; Lesaint et al., 2014).

In the current study, we highlight key differences between the coding properties of NAc neurons with different signaling profiles: excitatory versus inhibitory responses to a cue that predicts reward. While studies have shown that ∼50% of NAc neurons exhibit phasic excitations in response to a reward-associated cue, an additional 25–30% exhibit phasic inhibitions (Morrison et al., 2017). We were motivated by the observation that cue-evoked inhibitions, compared with excitations, are far less sensitive to dopaminergic manipulations such as dopamine receptor antagonists (du Hoffmann and Nicola, 2014). Therefore, we wondered whether inhibitions were less sensitive than excitations to behavioral differences thought to be related to different profiles of dopamine release (Flagel et al., 2011) – sign tracking versus goal tracking, and/or behavioral manipulations, such as extinction and reward devaluation, that have been shown to affect dopamine release in the NAc (Sunsay and Rebec, 2014; Aitken et al., 2016; Papageorgiou et al., 2016) .

We found that NAc cue-evoked inhibitions become more prevalent over the course of training on a Pavlovian conditioned approach task, and that inhibitions, like excitations, may encode behavioral vigor. On the other hand, we found that behavioral differences that are associated with profound changes in NAc excitatory responses do not affect inhibitory responses in a similar way. For example, phasic inhibitions associated with reward delivery, unlike excitations, are not different between sign trackers and goal trackers. Moreover, cue-evoked inhibitions, unlike many cue-evoked excitations, are not affected by extinction of the association between cue and reward. Finally, using a reward devaluation procedure that abolishes both sign tracking and goal tracking behaviors (Amaya et al., 2020), we show that cue-evoked excitations become profoundly attenuated, whereas cue-evoked inhibitions remain stable. Thus, as a population, cue-excited cells in the NAc closely track behavior; but cue-inhibited cells, at least once their response is established, do not.

### NAc neural signaling during acquisition of conditioned behavior

Cue-responsive neurons in the NAc, including both cue-excited and cue-inhibited cells, were present from the very first day of training. As we have previously reported (Gillis and Morrison, 2019), cue-excited cells do not increase their cue-evoked response over the course of the first training session, although sign trackers (but not goal trackers) show a pronounced decrease in reward-evoked excitation. The presence of cue-evoked excitations at the start of training is consistent with recent studies showing robust NAc dopamine release to novel cues – even when they are not (yet) associated with reward – which decreases as cues become familiar (Morrens et al., 2020; Kutlu et al., 2022).

We did not have a sufficient population of cue-inhibited neurons from sign trackers on the first day of training to evaluate whether their cue-evoked or reward-evoked inhibitions changed during early learning, although we did observe that at least some inhibitions were present from the earliest trials. Notably, however, unlike cue-evoked excitations (Gillis and Morrison, 2019), cue-evoked inhibitions increased in both intensity and representation across the population over the course of training. This implies that cue-inhibited NAc neurons exhibit plasticity related to the acquisition of ST and/or GT behaviors; this neural plasticity is unlikely to be related to changes in dopamine release, since inhibitions are largely insensitive to dopaminergic manipulation (du Hoffmann and Nicola, 2014). Recent evidence indicates that NAc cue-evoked excitations change in concert with behavior during instrumental learning in an NMDA receptor-dependent manner (Vega-Villar et al., 2019); additional experiments are needed to determine whether inhibitions also exhibit NMDAR-dependent plasticity.

We found that at least some cue-evoked inhibitions encode the vigor of subsequent ST and GT behavior, consistent with NAc cue-evoked inhibitions encoding such factors as motion onset latency during instrumental tasks (Morrison et al., 2017). The dual encoding of ST and GT behavior supports the view of the NAc as a hub for invigoration of behavior stemming from different forms of reward learning. Many authors have argued that ST is the result, at least in part, of a dopamine-dependent model-free learning process, and that GT results from a separate, non-dopamine dependent process that utilizes the predictive qualities of the cue without transferring incentive salience from reward to cue (Clark et al., 2012; Lesaint et al., 2014, 2015). The NAc appears to participate in promoting both types of motivated behavior via both excitatory and inhibitory responses to reward-associated cues.

### Divergent relationships of excitatory and inhibitory NAc neural signals with behavior

In the current study, we did not directly measure or manipulate dopamine; however, others have observed changes in dopamine release specific to sign trackers, including enhanced NAc dopamine release in response to a reward-predictive cue over the course of training, along with a reduced response to reward delivery (Flagel et al., 2011). Goal trackers, in contrast, did not show major changes in NAc dopamine release during learning. If this is the case in the current subject population, then cue-excited neurons in the NAc might reflect these differences in dopamine release: their response to reward delivery is substantially reduced over the course of training among sign trackers, but not goal trackers, as we have reported previously (Gillis and Morrison, 2019). We found that cue-inhibited cells in the NAc, on the other hand, do not follow this pattern: their reward-related responses do not diverge between sign tracker and goal tracker individuals. Thus, if there are differences in dopamine release between sign trackers and goal trackers, it does not seem to have a major impact on NAc inhibitory responses. This conclusion is in line with the observation that dopaminergic manipulations do not substantially impact NAc inhibitions, although they strongly affect excitations. Specifically, du Hoffmann and Nicola (2014) showed that blockade of either D1 or D2 receptors in the NAc led to a profound reduction in cue-evoked excitations, but not inhibitions; indeed, in some cases, dopamine receptor antagonism resulted in an increase in inhibitory responding, apparently resulting from an unmasking of inhibition that had been obscured by excitatory responses.

These results may also help explain our finding that NAc cue-evoked inhibitions, unlike excitations, are virtually unaffected by extinction of the cue-reward relationship. As we have previously shown (Gillis and Morrison, 2019), many neurons with cue-evoked excitations show a decrease in responding during extinction that corresponds closely to behavior. Notably, at the same time, a subset of neurons with cue-evoked excitations do not change their firing during extinction; we refer to these as “non-extinguishing” cells. In the current study, we found that nearly all cue-inhibited neurons may be categorized as non-extinguishing. Studies have shown that extinction of a Pavlovian conditioned cue correlates with reduced dopamine release in the NAc core (Sunsay and Rebec, 2014). which is likely a factor in the extinction of both behavior and NAc excitatory responses. In contrast, our data suggests that it does not affect cue-evoked inhibitory responses. Together with non-extinguishing excitatory responses, these signals might play a role in maintaining a latent association between cue and reward after extinction, contributing to such processes as spontaneous recovery and reinstatement (Todd et al., 2014).

Similarly, we found that NAc cue-evoked excitations are drastically reduced by a reward devaluation procedure, while cue-evoked inhibitions are virtually unaffected. Reward devaluation has been shown to reduce NAc dopamine release to a cue associated with the devalued reward (Aitken et al., 2016; Papageorgiou et al., 2016). Importantly, the suppression of cue-evoked excitatory signaling is apparent from the earliest trials following devaluation, and does not require the subject to experience any pairings of the cue with the devalued reward, similar to changes in behavior (Murray and Izquierdo, 2007; West and Carelli, 2016) and dopamine release (Aitken et al., 2016). Future experiments could investigate whether and when the devaluation effect on NAc excitatory cue encoding requires input from the orbitofrontal cortex (OFC) and/or basolateral amygdala; many studies have shown that these areas are essential parts of a neural circuit that links a cue to the new, lower value of its associated reward (Murray and Izquierdo, 2007; McDannald et al., 2014).

To our knowledge, only one other study has examined the impact of outcome devaluation on the activity of individual neurons in the NAc. West and Carelli (2016) trained rats on an instrumental task and used selective satiation to devalue the reward associated with a lever cue. They found that reward devaluation decreased cue representation, defined as the proportion of neurons exhibiting any cue response, in the NAc shell but not the NAc core. In the current study, we too saw only a small reduction in the number of cue-responsive neurons after reward devaluation. However, we also observed a large shift in the proportions of excitatory versus inhibitory responses: in our dataset, inhibitory responses became relatively overrepresented after reward devaluation. Surprisingly, West and Carelli (2016) also found little or no decrement in the magnitude of excitatory responses in the NAc core. There are several differences in approach that might contribute to the apparent discrepancy. Both the use of an instrumental task with multiple levers (i.e., multiple possible approach targets), and the use of a more temporary method, selective satiety, to devalue one of two possible rewards, might account for differences in cue encoding in the NAc core. Indeed, there is evidence that NAc signaling is not especially sensitive to short-term changes in cue-reward contingency (Morrison and Nicola, 2014; Morrison et al., 2017). Finally, the use of reinforcer devaluation performed outside of the task context might have contributed to relatively weak effects on signaling in the NAc core.

The behavioral effects of reward devaluation in the current study contrast with some of our earlier findings (Morrison et al., 2015; Rode et al., 2019), as well as those of others (Nasser et al., 2015; Patitucci et al., 2016; Vandaele et al., 2017), that ST behavior and/or sign tracker individuals are relatively insensitive to changes in reward value. These findings have been complicated by other reports (Derman et al., 2018; Amaya et al., 2020) showing that sign tracking can be sensitive to reward devaluation under certain circumstances. In particular, Amaya et al. (2020) reported that ST behavior was sensitive to devaluation that took place in the testing context, but not outside of it. In the current study, we used an in-context devaluation along with multiple (four to five) pairings of the reward with LiCl and found that this procedure profoundly reduced both ST and GT behavior. It is important to note that these results are not inconsistent with the idea that sign tracking is less sensitive to devaluation than goal tracking. Multiple groups have shown that GT behavior, in contrast to ST, is affected by even “weak” forms of reward devaluation, including devaluation that takes place in a different context (Morrison et al., 2015) and/or devaluation that is accomplished by selective satiation (Patitucci et al., 2016; Kochli et al., 2020; Keefer et al., 2022) rather than taste aversion conditioning.

Evidence from multiple studies indicates that sign tracking and goal tracking are the products of distinct, but partially overlapping, behavioral and neural processes. Within the NAc, ST and GT individuals show stark differences in dopamine dependence and patterns of dopamine release (Flagel et al., 2011; Saunders and Robinson, 2012), as well as differences in the encoding of reward over training (Gillis and Morrison, 2019), but these differences are observed in some neurons (cue-excited cells) and not others (cue-inhibited cells); moreover, cue-evoked responses seem to promote vigorous behavioral responding in both ST and GT modes. Further dissection of the neural circuits that support sign tracking, goal tracking, or both may shed light on their emerging relationship with drug abuse and addiction vulnerability.

## Synthesis

Reviewing Editor: Mark Laubach, American University Decisions are customarily a result of the Reviewing Editor and the peer reviewers coming together and discussing their recommendations until a consensus is reached. When revisions are invited, a fact-based synthesis statement explaining their decision and outlining what is needed to prepare a revision will be listed below. The following reviewer(s) agreed to reveal their identity: Donna Calu, Benjamin Saunders.

Please see the reviewer’s comments below and revise your manuscript accordingly. Also, please consider adding a visual abstract, which helps with attracting readers to your paper. Thank you for sending your study to eNeuro.

Both reviewers raised concern about your data analysis. Reviewer 1 asked about your extensive use of the Wilcoxon Test, and if a two-way ANOVA would be better for some data summaries (e.g. Figure 7B). My reading of your methods section raises concern about using a 10 ms bin with 5-bin moving average smoothing. Can you comment on your rationale for this bin size and also show if results vary with other bin sizes? 10, 25, 50 ms without smoothing? Also, you should comment oon the appropriateness of your data for ANOVA, specifically if spike counts should be transformed to stabilize variance before using ANOVA (e.g. log transform) and if spike count distributions overlap for the Wilcoxon test.

Reviewer #1

The authors characterize NAc core firing responses during autoshaping, extinction, and devaluation conditions. The manuscript establishes that NAc core neurons - whose firing reduces to lever cues - increase these inhibitions over time (unlike excitatory responses which are established on the 1st day of training) in a PLA task in ST and GT. Furthermore, unlike excitatory responses in the NAc, which are generally suppressed by extinction conditions and by devaluation, the inhibitory responses remain stable during extinction and inhibition. They are also similar between ST and GT, though may persist for longer in ST during extinction. This data fits in with a body of work showing that dopamine responses in NAc are correlated with behavior and excitatory neural responses and differ between tracking types. It is interesting because inhibitory cells in NAc do not follow this same pattern. This data adds color to our understanding how NAc functions during presentation of cues which no longer engender responding, advancing the field.

In general, the study adds information regarding how NAc may function similarly and differently between sign and goal trackers, as well as how the NAc encodes cues whose associative meaning with rewards have changed due to extinction or devaluation. Some of the figures and data need some clarification, see below.

Minor Comments

- Figures need Titles or better labeling

- There is no strong indication from primary data that the cells are encoding vigor in any consistent way in this study, though some cells’ activity appears to correlate with vigor. Are the statistics in figure 3 corrected for multiple comparisons? (IE could these cells have been statistically related to vigor by chance given the large number of cells examined)?

- Figure 4B has no statistics associated with it, yet it is referred to in text as the last session is stronger inhibition - this strength of inhibition is not obviously statistically different in the image. How is this strength quantified? The statistics referred to in table are for 4C, not 4B.

- In Figure 6D, the inhibition appears slight, when averaged across all inhibitory cells. Perhaps this is because peak inhibition is at different times for different cells. However, this is not the case during regular training sessions, which show a relatively large decrease in activity at cue onset. Is there a difference in the level of inhibition between regular and extinction conditions (even though across extinction bins, inhibition appears constant). It appears data may have been combined across 2 extinction sessions - are there differences in inhibition between the extinction sessions?

Reviewer #2

In this study, nucleus accumbens core neurons are recorded during a Pavlovian conditioned approach task. The dataset builds from an earlier one from this group using the same approach. Here, neurons showing excitations and those showing inhibitions in response to Pavlovian cue presentations were seen, as with other reports. The emergence of sign and goal tracking behaviors was measured. Overall, they find that cue-excited neurons, but not cue-inhibited neurons, change their firing patterns over the course of Pavlovian learning and extinction.

The connection to dopamine signaling referenced throughout the manuscript is perhaps too strong, given that no dopamine measurements or manipulations are made here. The logic of the interpretation of the effects of dopamine on excitatory and inhibitory signals in the accumbens makes sense overall, but I think it’s a bit of a reach to directly related each of the subtle effects here to the dopamine signaling. I don’t think it can be assumed that the rats tested here have unique patterns of dopamine signaling, for example. And the connection between differences in dopamine signaling in sign and goal trackers from other studies is assumed to be the same here, which isn’t a certainty without separate confirmation.

It would be helpful to see the neural measures split by goal/sign tracker group. Do some rats have greater proportions of different neural patterns? The connection between the excitations/inhibitions story and the sign and goal tracking is a bit loose to me.

Overall I’m finding the figures hard to parse - maybe they need more labels or some kind of delineation of what statistical comparisons are being made. You can’t tell just from the figures what most of the conclusions are.

Figure 6 - It makes sense to show the example neuron pattern but it’s not appropriate to run statistics on a single neuron’s activity.

Overall the inhibitions are smaller - less deflection in firing rate - compared to the excitations. Is it possible that there are just limitation to how different the inhibition signals can be? What would be a situation where an inhibition would definitely grow stronger or weaker, for example?

The discussion could use focusing and shortening.

It would be helpful to a bit more directly delineate what specific data points and conclusions are new to this paper versus the 2019 paper from the same lab. Since this paper also reuses most of the data from the earlier paper and restates several of the same conclusions.

## Author Response

We thank the reviewers for their feedback and suggestions for improvement. We have endeavored to address each of the reviewers’ points in our revision of the paper and/or in our specific responses below (in italics).

Synthesis Statement for Author (Required):

Please see the reviewer’s comments below and revise your manuscript accordingly. Also, please consider adding a visual abstract, which helps with attracting readers to your paper. Thank you for sending your study to eNeuro.

Both reviewers raised concern about your data analysis. Reviewer 1 asked about your extensive use of the Wilcoxon Test, and if a two-way ANOVA would be better for some data summaries (e.g. Figure 7B).

--We are slightly puzzled by this comment, as we don’t see this concern articulated by Reviewer 1 below; nevertheless, we are happy to add two-way ANOVA as an additional way to look at the data, specifically in Figures 6E,F and 7B,C. We have now added two-way ANOVA statistics to the text and Table 1 for these figures. For Figure 6, a two-way ANOVA with factors of trial number (continuous) and sign tracker vs. goal tracker supports the conclusion that, as a population, cue-evoked excitations, but not inhibitions, extinguish along with behavior during extinction (main effect of trial number). For Figure 7, a two-way ANOVA with factors of trial number (continuous) and devaluation status supports the conclusion that excitations, but not inhibitions, are dramatically suppressed following reward devaluation (interaction of devaluation status and trial number).

For these figures, we have decided to keep using the Wilcoxon rank sum test for pairwise comparisons between groups for specific trial bins (asterisks in Fig. 6E,F and Fig. 7B). This makes sense when we want a “snapshot” comparison of activity at a particular time point (e.g. the key earliest time point following devaluation). (Notably, the traditional post-hoc tests don’t apply when one of the factors, trial number, is continuous.) We believe the Wilcoxon is preferable to a t-test, overall, because it doesn’t make assumptions about the distribution of the data (see further discussion below).

My reading of your methods section raises concern about using a 10 ms bin with 5-bin moving average smoothing. Can you comment on your rationale for this bin size and also show if results vary with other bin sizes? 10, 25, 50 ms without smoothing?

--We agree that choices of bin size and smoothing can be a concern when presenting population-averaged PSTH data. This is one reason we include non-smoothed heat plots (Fig. 1C-F) showing Z-scored activity for each cell that is included in subsequent analyses. We also include raw data for several example cells (e.g., Fig. 3A-D). These are also the same parameters we use for population PSTHs in our previous papers, including Gillis & Morrison, 2019, making it easier to compare the current data with results from earlier reports.

For population PSTH figures, we chose this particular bin size and smoothing parameter because it increases legibility (by smoothing) without sacrificing detail (as with a larger bin size). For illustration, here are the population PSTHs from Figure 4 shown without smoothing:

And here are the same population PSTHs using a 25ms bin (unsmoothed):

Any of these versions is fine, but none would change the overall conclusions. Therefore, we have opted to keep the current versions of the figures. Finally, please note that none of our statistical analyses are performed on smoothed data.

Also, you should comment on the appropriateness of your data for ANOVA, specifically if spike counts should be transformed to stabilize variance before using ANOVA (e.g. log transform) and if spike count distributions overlap for the Wilcoxon test.

--Thank you for the suggestion! We think that the data from Figures 6E,F and 7B,C are reasonably suitable for ANOVA. Although we generally prefer to use nonparametric tests when possible, we are happy to take the reviewers’ advice. After careful consideration, we do not believe log transformation is necessary in this case: the data is already Z-scored and we found that log transformation makes little difference to the results of any of the ANOVAs that are now included. In general, we would prefer less-processed data where appropriate, so we will omit the log transformation for now; however, if reviewers feel strongly, we can go back and apply the transformation prior to ANOVA.

We are not sure what information reviewers are requesting regarding suitability of data for the Wilcoxon, but we understand that “ties” between data points can be a concern when using a rank-based test. We should point out that the Wilcoxon is being performed on Z-scored data, which is normalized to a different mean and standard deviation for each cell, so data from different populations of cells is very unlikely to contain numerically identical values. Therefore, ties are unlikely to be an issue. Moreover, Z-scored response data generally fails tests of normality, so the Wilcoxon is preferable to parametric alternatives.

Reviewer #1

The authors characterize NAc core firing responses during autoshaping, extinction, and devaluation conditions. The manuscript establishes that NAc core neurons - whose firing reduces to lever cues - increase these inhibitions over time (unlike excitatory responses which are established on the 1st day of training) in a PLA task in ST and GT. Furthermore, unlike excitatory responses in the NAc, which are generally suppressed by extinction conditions and by devaluation, the inhibitory responses remain stable during extinction and inhibition. They are also similar between ST and GT, though may persist for longer in ST during extinction. This data fits in with a body of work showing that dopamine responses in NAc are correlated with behavior and excitatory neural responses and differ between tracking types. It is interesting because inhibitory cells in NAc do not follow this same pattern. This data adds color to our understanding how NAc functions during presentation of cues which no longer engender responding, advancing the field.

In general, the study adds information regarding how NAc may function similarly and differently between sign and goal trackers, as well as how the NAc encodes cues whose associative meaning with rewards have changed due to extinction or devaluation.

We thank the reviewer for their supportive comments!

Some of the figures and data need some clarification, see below.

Minor Comments

- Figures need Titles or better labeling

--We apologize for the confusion: the titles are found with the figure legends. We have endeavored to improve our figure labeling, including in-figure legends for every color or line type.

- There is no strong indication from primary data that the cells are encoding vigor in any consistent way in this study, though some cells’ activity appears to correlate with vigor. Are the statistics in figure 3 corrected for multiple comparisons? (IE could these cells have been statistically related to vigor by chance given the large number of cells examined)?

--We certainly understand the reviewer’s concern, but no, the p-values used to determine significance for individual cells in Figure 3 are not corrected for multiple comparisons. Such a correction (e.g., Bonferroni) would not be practical because there is a p-value “floor” given the limited number of observations available for each cell (i.e., trials with low vs. high vigor). If the reviewer wishes, we can eliminate the indications for “significance” for individual cells (blue bars in Fig. 3E,F). However, we would prefer to keep them for easy comparability with the equivalent plot in Gillis & Morrison, 2019.

Either way, we would suggest that the key statistic is not the significance or non-significance of the “vigor index” for individual cells; rather, it is whether the population of ROC values is shifted in such a way that suggests that the population of cells encodes vigor. We previously demonstrated such a shift among cue-excited cells, especially for magazine entry latency, in Gillis & Morrison, 2019. Figure 3e and its associated statistics allow the reader to compare the current data set with the subset shown in that previous paper and adds the complementary plot for cue-inhibited neurons (Fig. 3f). Although neither population is significantly shifted away from 0.5 in the current data set, it lets the reader see that the distributions for cue-excited vs. cue-inhibited neurons are skewed in opposite directions (i.e., in the direction of more intense excitations and inhibitions for trials with greater vigor).

- Figure 4B has no statistics associated with it, yet it is referred to in text as the last session is stronger inhibition - this strength of inhibition is not obviously statistically different in the image. How is this strength quantified? The statistics referred to in table are for 4C, not 4B.

--We thank the reviewer for pointing out this oversight in our statistics, as well as the error in the table! The reviewer is correct in their intuition that the strength of inhibitions, on average, does not significantly increase over the course of training (except in certain specific time windows, but we think it is more meaningful to use customized time windows for each neuron). We have changed the text to reflect this, removing any reference to increasing strength of inhibitions across training. We don’t think this substantially changes our conclusions, as the number/proportion of inhibited cells does increase over the course of training. We have corrected the table and added the tests related to Fig. 4A and 4B.

- In Figure 6D, the inhibition appears slight, when averaged across all inhibitory cells. Perhaps this is because peak inhibition is at different times for different cells. However, this is not the case during regular training sessions, which show a relatively large decrease in activity at cue onset. Is there a difference in the level of inhibition between regular and extinction conditions (even though across extinction bins, inhibition appears constant).

--We agree that the level of inhibition appears relatively small in Fig. 6D, but this is an artifact of (1) a smaller population of inhibited cells recorded during extinction (n = 22 vs. n = 47 for the last day of training) and (2) variability in the timing of peak inhibition, as the reviewer suggests. (Note also that the population PSTH curves in this figure are only from the first 5 vs. last 5 trials of extinction, which increases the variability as well.) In response to the reviewer’s concern, we tested whether there is a difference in the overall inhibitory response between the final day of training and the extinction session. There was no significant difference when measured in a 500 ms time window after cue onset (p = 0.62, Wilcoxon rank sum).

It appears data may have been combined across 2 extinction sessions - are there differences in inhibition between the extinction sessions?

We thank the reviewer for pointing out this ambiguity. Although some rats experienced two extinction sessions, all analyses shown use data from the first extinction session only. We now make this clear in the Methods section.

Reviewer #2

In this study, nucleus accumbens core neurons are recorded during a Pavlovian conditioned approach task. The dataset builds from an earlier one from this group using the same approach. Here, neurons showing excitations and those showing inhibitions in response to Pavlovian cue presentations were seen, as with other reports. The emergence of sign and goal tracking behaviors was measured. Overall, they find that cue-excited neurons, but not cue-inhibited neurons, change their firing patterns over the course of Pavlovian learning and extinction.

--The connection to dopamine signaling referenced throughout the manuscript is perhaps too strong, given that no dopamine measurements or manipulations are made here. The logic of the interpretation of the effects of dopamine on excitatory and inhibitory signals in the accumbens makes sense overall, but I think it’s a bit of a reach to directly related each of the subtle effects here to the dopamine signaling. I don’t think it can be assumed that the rats tested here have unique patterns of dopamine signaling, for example. And the connection between differences in dopamine signaling in sign and goal trackers from other studies is assumed to be the same here, which isn’t a certainty without separate confirmation.

We absolutely take the reviewer’s point, and we thank the reviewer for the constructive criticism. We do not measure or manipulate dopamine in the current study, and we certainly don’t want to mislead or overreach in our conclusions. To that end, we have moderated our language in the abstract, introduction, and discussion regarding the connections between the current findings and earlier studies that measured or manipulated dopamine. For example, the final sentence of the abstract, which previously read:

“Overall, these findings converge with existing evidence that cue-excited neurons in NAc, but not cue-inhibited neurons, are profoundly sensitive to changes in dopamine release, whether due to dopamine-dependent reward learning, extinction, or outcome devaluation.”

Now reads:

“Overall, these findings converge with existing evidence that cue-excited neurons in NAc, but not cue-inhibited neurons, are profoundly sensitive to the same behavior variations that are often associated with changes in dopamine release.”

Furthermore, we have added an explicit caveat in the discussion, including a sentence starting “In the current study, we did not directly measure or manipulate dopamine...” prominently placed right after the subheading “Divergent relationships of excitatory and inhibitory NAc neural signals with behavior” (which used to read “Divergent relationships of NAc neural signals with dopamine release”).

It would be helpful to see the neural measures split by goal/sign tracker group. Do some rats have greater proportions of different neural patterns? The connection between the excitations/inhibitions story and the sign and goal tracking is a bit loose to me.

We thank the reviewer for the excellent question! Here is the breakdown by sign tracker vs. goal tracker for the final training session: among sign trackers (n=5), there were 29 (57%) cue-excited cells, 14 (27%) cue-inhibited cells, and 8 (16%) cells with no cue response (total 51 cells). Among goal trackers (n=6), there were 50 (42%) cue-excited cells, 35 (30%) cue-inhibited cells, and 33 (28%) cells with no cue response (total 118 cells). Overall, these proportions are not significantly different from each other (p = 0.14, chi-square test), although there is a bit of a trend towards fewer cue-excited cells in goal trackers (p = 0.08, chi-square test for “excited” vs. “non-excited” categories).

Interestingly, this was more than a trend on the first day of training, although fewer cells were recorded overall: among sign trackers (n=5), there were 25 (78%) cue-excited cells, 2 (6%) cue-inhibited cells, and 5 (16%) cells with no cue response (total 32 cells). Among goal trackers, there were 20 (33%) cue-excited cells, 12 (20%) cue-inhibited cells, and 28 (47%) cells with no cue response (total 60 cells). These proportions are significantly different (p < 0.001, chi-square test using either two or three categories). Therefore, we have decided to add this data to Figure 4. I would hesitate to read too much into these differences, given the small sample size, but it never hurts to provide more of the raw data to the reader. We made sure to interpret the data with caution in the text.

Overall I’m finding the figures hard to parse - maybe they need more labels or some kind of delineation of what statistical comparisons are being made. You can’t tell just from the figures what most of the conclusions are.

--In response to this concern and that of Reviewer 1, we have endeavored to add more legends and labels within the figures. We have also added some relevant statistics to the figure legends as well as the text. We think that having the figure titles and figure legends adjacent to the figures will alleviate this issue as well.

Figure 6 - It makes sense to show the example neuron pattern but it’s not appropriate to run statistics on a single neuron’s activity.

--We must respectfully disagree. It is common practice to run an analysis, such as an ANOVA or GLM, individually on each neuron’s activity - often with the intent of showing the population distribution of coefficients or other indices (as in Figure 3C,D) - so what’s the harm in providing such a statistic for one neuron out of the population? More specifically, we provide the one-way ANOVA results for the example neurons in Fig. 6A,B in order to place them in the context of the “extinguishing” vs. “non-extinguishing” neuron populations defined in Gillis & Morrison, 2019. We now make this more explicit in the corresponding text in the results.

Overall the inhibitions are smaller - less deflection in firing rate - compared to the excitations. Is it possible that there are just limitation to how different the inhibition signals can be? What would be a situation where an inhibition would definitely grow stronger or weaker, for example?

--We thank the reviewer for the thoughtful questions. These are issues that we have thought and written about quite a bit (see Morrison et al., 2017). The reviewer is correct: given the typical baseline firing rates of NAc neurons, there is a smaller dynamic range available for inhibitory responses compared with excitatory responses. This is somewhat mitigated by the use of Z-scoring to transform inhibitions relative to baseline firing rate; but it is nevertheless more difficult, statistically, to detect encoding of any given parameter (e.g., action latency in Fig. 3). However, I don’t think this presents a problem for any of the conclusions of the paper, and it doesn’t sound like the reviewer is actually concerned about that. Rather, the reviewer asks about limitations on “how different the inhibition signals can be.”

Reading between the lines, I think the reviewer is mainly concerned about the lack of difference in inhibitory signals shown in some of the population PSTH figures (in particular, I’m guessing Fig. 5D and Fig. 6D). As we address in our response to Reviewer 1, the relatively weak-looking signals in Fig. 6D are the result of (1) a smaller data set for extinction and (2) only 5 trials included in each curve, both amplifying variability among the times of peak inhibition. The relatively weak-looking signals in Fig. 5D are because of variable responses to reward among the cue-inhibited neural population (most cue-inhibited cells are also reward-inhibited, but not all, and the timing can vary). However, whenever we show a population PSTH and make claims about differences (or lack of such) in response, we separately analyze the distribution of cue- or reward-evoked responses in customized time windows that capture the actual onset/offset of inhibition. Then, by using a Wilcoxon rank sum test, which is insensitive to the range of values being compared, we are able to determine whether one distribution is significantly larger/smaller than the other. We are confident that this analysis, combined with the confidence intervals in the PSTH, is a robust test of the difference (or lack of difference) in inhibition between two populations (or a single population at different time points).

The discussion could use focusing and shortening.

--We have revised the discussion from 2851 words to just over 2000 words.

It would be helpful to a bit more directly delineate what specific data points and conclusions are new to this paper versus the 2019 paper from the same lab. Since this paper also reuses most of the data from the earlier paper and restates several of the same conclusions.

--We appreciate the reviewer’s concern. We generally point out in the text when we are reproducing a result from the previous, smaller data set from the 2019 paper. To address the concern, we have now added a note in each figure legend pointing out which panels are essentially reproductions of similar figures from the 2019 paper (and direct the reader to the specific figure panel in that paper).

References cited in this response to reviewers:

Gillis ZS, Morrison SE (2019) Sign tracking and goal tracking are characterized by distinct patterns of nucleus accumbens activity. eNeuro 6.

Morrison SE, McGinty VB, du Hoffmann J, Nicola SM (2017) Limbic-motor integration by neural excitations and inhibitions in the nucleus accumbens. J Neurophysiol 118:2549-2567.
